# The circulating proteomic signature of alcohol-associated liver disease

**DOI:** 10.1172/jci.insight.159775

**Published:** 2022-07-22

**Authors:** Jay Luther, Augustin G.L. Vannier, Esperance A. Schaefer, Russell P. Goodman

**Affiliations:** Massachusetts General Hospital Alcohol Liver Center, Division of Gastroenterology, Massachusetts General Hospital, Boston, Massachusetts, USA.

**Keywords:** Hepatology, Hepatitis, Proteomics

## Abstract

Despite being a leading cause of advanced liver disease, alcohol-associated liver disease (ALD) has no effective medical therapies. The circulating proteome, which comprises proteins secreted by different cells and tissues in the context of normal physiological function or in the setting of disease and illness, represents an attractive target for uncovering novel biology related to the pathogenesis of ALD. In this work, we used the aptamer-based SomaScan proteomics platform to quantify the relative concentration of over 1300 proteins in a well-characterized cohort of patients with the spectrum of ALD. We found a distinct circulating proteomic signature that correlated with ALD severity, including over 600 proteins that differed significantly between ALD stages, many of which have not previously been associated with ALD to our knowledge. Notably, certain proteins that were markedly dysregulated in patients with alcohol-associated hepatitis were also altered, to a lesser degree, in patients with subclinical ALD and may represent early biomarkers for disease progression. Taken together, our work highlights the vast and distinct changes in the circulating proteome across the wide spectrum of ALD, identifies potentially novel biomarkers and therapeutic targets, and provides a proteomic resource atlas for ALD researchers and clinicians.

## Graphical abstract

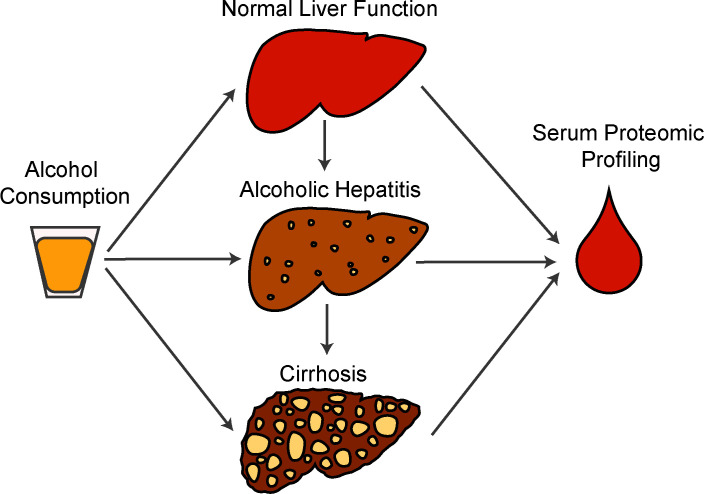

## Introduction

Alcohol-associated liver disease (ALD) represents a wide spectrum of disease, ranging from bland steatosis with limited liver-related mortality to cirrhosis and hepatocellular carcinoma. Alcohol-associated hepatitis (AH), the most severe form of ALD, is characterized by the rapid onset of jaundice in the setting of heavy drinking (1). In part because of its poorly understood pathogenesis, AH carries a markedly high 90-day mortality rate of 30% to 50% (2), a mortality rate that rivals or exceeds that of many forms of metastatic cancer (3–5). Despite intense clinical efforts to identify therapeutic approaches to treat ALD, there are currently no medical therapies for this condition that provide a convincing, durable clinical benefit (2). It is likely that the clinical management of ALD would be aided by the identification of quantitative, clinically relevant biomarkers of disease progression, which could in turn provide insight into the development of new potential therapeutic targets.

The circulating proteome offers an attractive target for both biomarker and therapeutic target discovery. Blood is easily sampled and contains the most complete version of the human proteome among any tissues (6), which both reflects and influences physiological and pathological states in different tissues and organs. A major technical challenge in characterizing the human plasma proteome has been its dynamic range, which exceeds 10 orders of magnitude in concentration (6), which far surpasses the dynamic range of any untargeted proteomic methods. The aptamer-based, proteomic platform SomaScan overcomes this limitation through the use of protein-specific aptamers that can simultaneously quantify over 1000 proteins of vastly different concentrations and has been previously used to characterize the circulating proteome in a number of metabolic and inflammatory diseases (7–9).

In this work, we leverage access to a well-characterized cohort of patients with the wide spectrum of ALD and report the application of the SomaScan platform to characterize the relative quantities of over 1300 circulating proteins in all stages of ALD. Our data illustrate marked changes occur in the circulating proteome of patients with ALD, and this protein dysregulation correlates with the severity of disease state and is particularly pronounced in those with severe AH. Notably, we show that the proteomic signature of severe AH is present even in heavy drinkers without clinical evidence of liver disease, suggesting that, rather than severe AH representing a distinct pathological entity, it is the severe manifestation of a pathological process present in all stages of ALD. Our proteomic data identify hundreds of potentially promising new biomarkers of ALD, including growth differentiation factor 15 (GDF15) and thrombospondin 2 (THBS2), and provide a large data set for future research projects.

## Results

### Proteomic evaluation across the wide spectrum of ALD.

**Figure 1:**
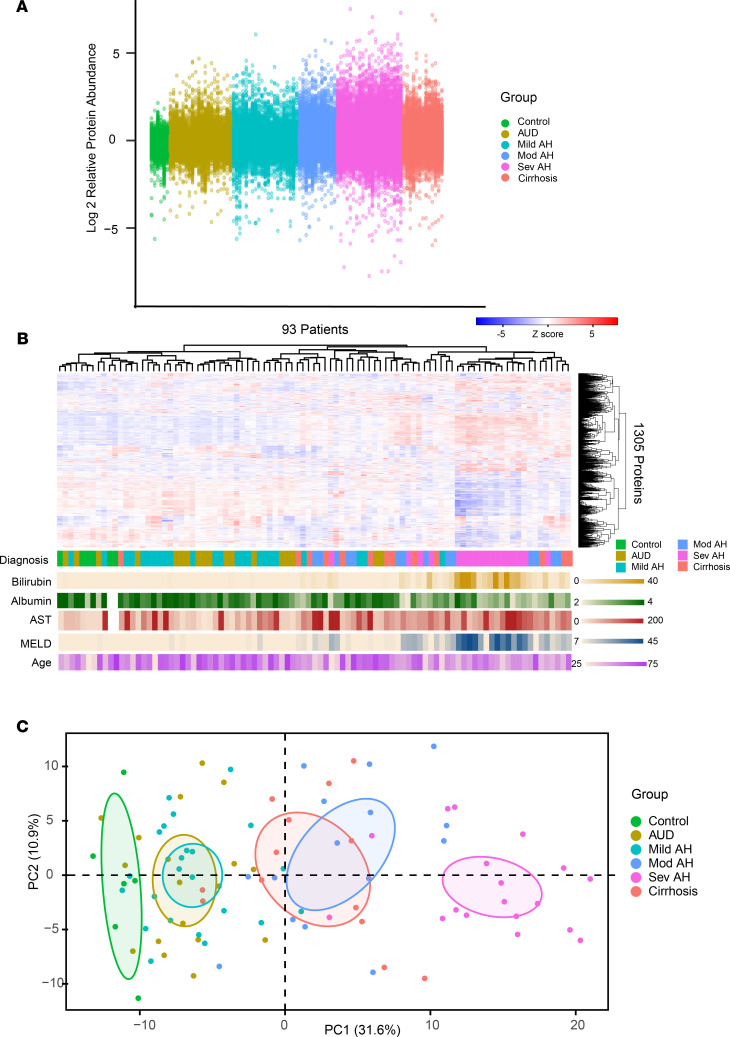
The proteomic signature of ALD. (**A**) Relative log_2_ fold abundance of each of 1305 proteins for each patient (*n* = 93) by clinical diagnosis. (**B**) Cluster dendrogram of patients versus relative protein abundance with clinical diagnosis and clinical characteristics. (**C**) PCA of relative protein abundance grouped by clinical diagnosis.

Table 1 summarizes the clinical characteristics of the study participants. Of the 1305 proteins measured in our study, we found roughly half (606 proteins) differed significantly (Bonferroni-adjusted *P* < 0.01) between groups (Supplemental Table 1; supplemental material available online with this article; https://doi.org/10.1172/jci.insight.159775DS1), with marked global alterations in protein abundance, particularly in patients with severe AH (Figure 1A). Patients with severe AH were additionally distinct in both unsupervised hierarchical clusters (Figure 1B) and principal component analysis (PCA, Figure 1C).

### Proteomic dysregulation correlates with the severity of AH.

**Figure 2:**
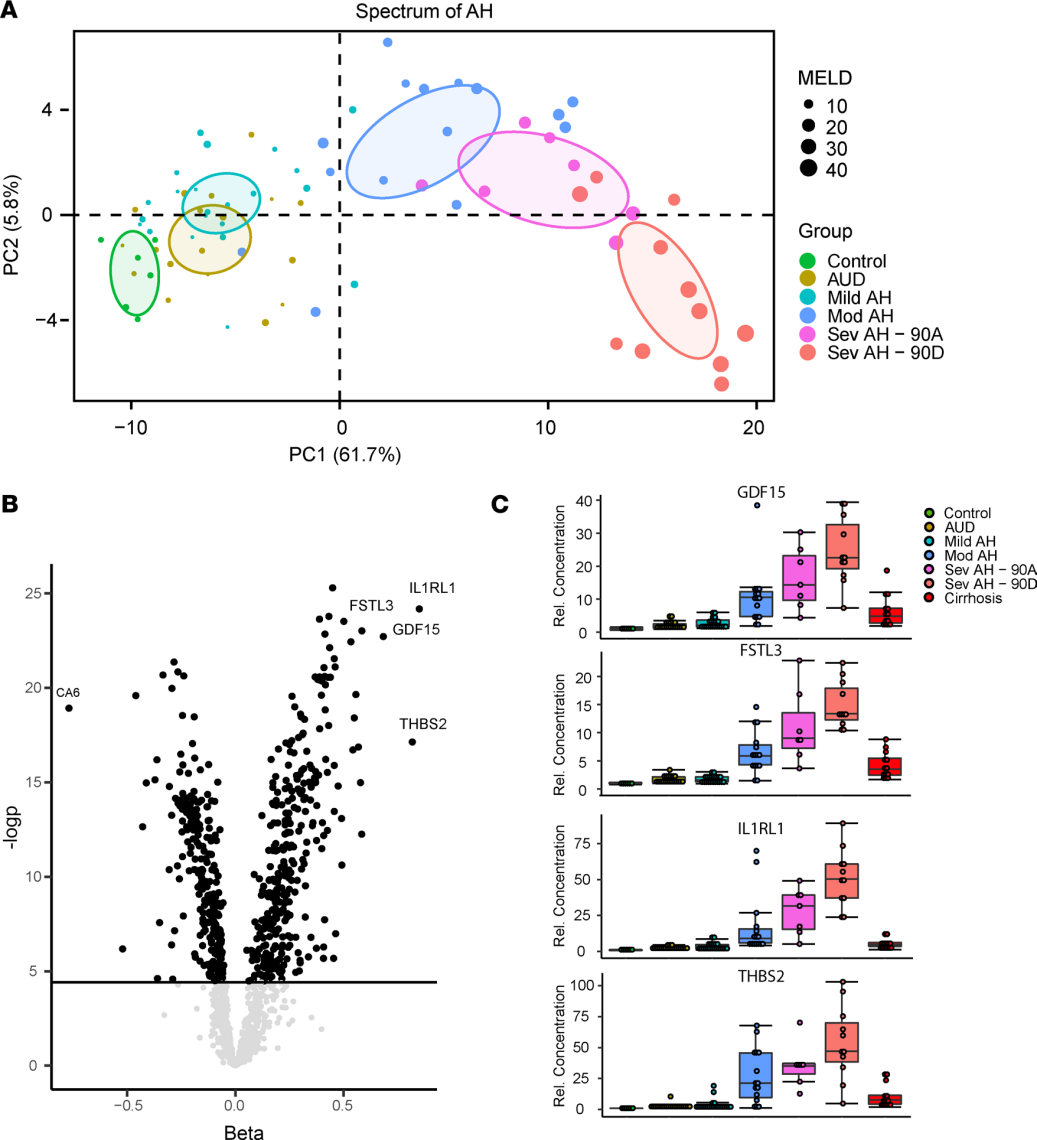
Proteomic dysregulation correlates with severity of AH. (**A**) PCA of significantly changing proteins (Bonferroni-corrected 1-way ANOVA) in the spectrum of AH. (**B**) Volcano plot of protein β and –log*P* values from the linear model. (**C**) Protein abundance by clinical group for GDF15, FSTL3, IL1RL1, and THBS2. Data are shown as mean ± SEM.

To better characterize the proteomic signature of AH, we focused our analyses on the spectrum of AH (control, alcohol use disorder [AUD], and mild, moderate, and severe AH) and further divided the severe AH group into those who were dead or alive after 90 days. PCA of this subgroup demonstrated the separation of patients by progressive severity of disease along PC1 (Figure 2A), which contained the majority of variance in proteins that differed significantly in this group (62%).

To identify proteins whose abundance increased with severity of AH, we performed 2 analyses. First, we identified those proteins contributing most to variance along PC1 (Table 2, left column). Second, we used a linear regression model adjusting for age, sex, and BMI, while modeling AH stage as an ordinal variable (Table 2, right column). Both approaches yielded similar results, identifying hundreds of proteins significantly associated with progressive severity of AH (Figure 2B). Among the most marked associations were THBS2, interleukin-1 receptor-like 1 (IL1RL1), follistatin-like 3 (FSTL3), and GDF15 (Figure 2C), which displayed marked and progressive changes in abundance along the spectrum of AH. Interestingly, patients with cirrhosis displayed an intermediate phenotype between patients with severe AH and controls, despite the fact that many patients with severe AH have significant fibrosis (10).

We observed approximately as many proteins increased in abundance (55%) as decreased in abundance (45%) in patients with severe AH (Supplemental Table 1), suggesting that the proteomic signature of severe AH was not solely due to the decreased hepatic synthetic function, which is commonly seen in severe liver disease. We further found no difference in self-reported weekly alcohol consumption by noncontrols (Supplemental Figure 1), suggesting differences in the proteomic signature by disease state were not directly attributable to differences in recent alcohol intake.

As validation of our measurements, we performed ELISA-based protein quantification on THBS2, IL1Rl1, FSTL3, and GDF15 in a subset of our initial cohort and in our validation cohort. ELISA measurements in both cohorts had excellent agreement with our SomaScan measurements in our initial cohort (Supplemental Figure 2).

### The proteomic signature of AH is detectable in patients with AUD and normal liver test results.

**Figure 3:**
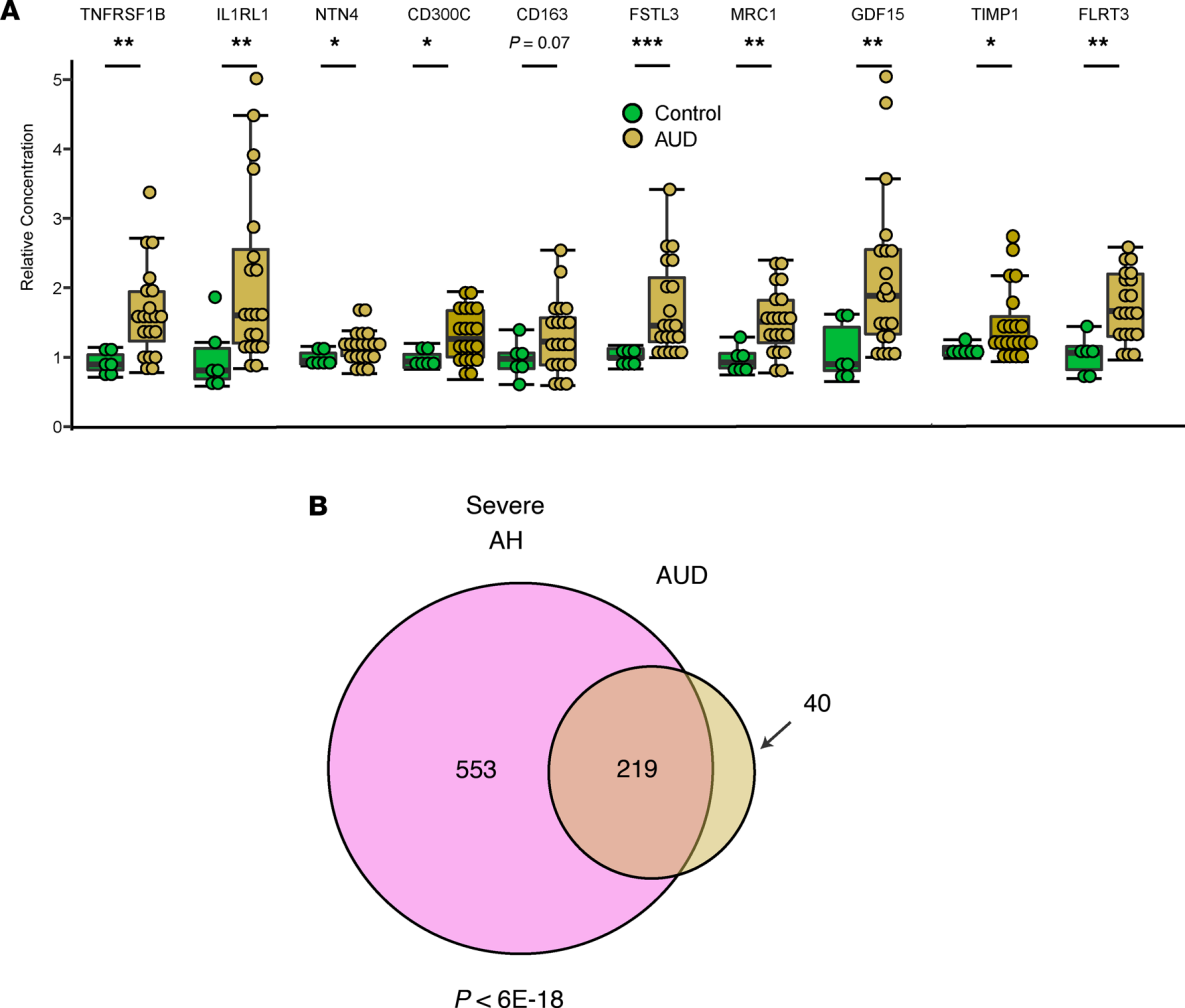
The proteomic signature of AH can be detected in heavy drinkers without clinically apparent liver disease. (**A**) Relative abundance of top 10 proteins associated with AH severity in patients with AUD versus controls. Data are shown as mean ± SEM, 1-tailed Mann-Whitney *U* test. *P < 0.05, **P < 0.01. (**B**) Overlap of nominally significantly altered proteins in severe AH versus AUD, with overlap *P* from hypergeometric test.

We examined the relative abundance of the top 10 proteins associated with severe AH in patients with AUD, who were defined as heavy drinkers admitted to the hospital for medically supervised withdrawal but who had normal liver test results and no clinical or laboratory evidence of liver disease. We were able to detect elevations in nearly all (9/10) proteins in this cohort (Figure 3A). Furthermore, the signature of proteins elevated in patients with AH and patients with AUD significantly overlapped (*P* < 6 × 10^–18^, hypergeometric test, Figure 3B). Together, these data indicate that the proteomic signature of severe AH was present in patients with heavy drinking but not overt liver disease, though to a much less significant elevation.

### The proteomic signature of 90-day transplant-free survival with severe AH highlights antimicrobial response.

**Figure 4:**
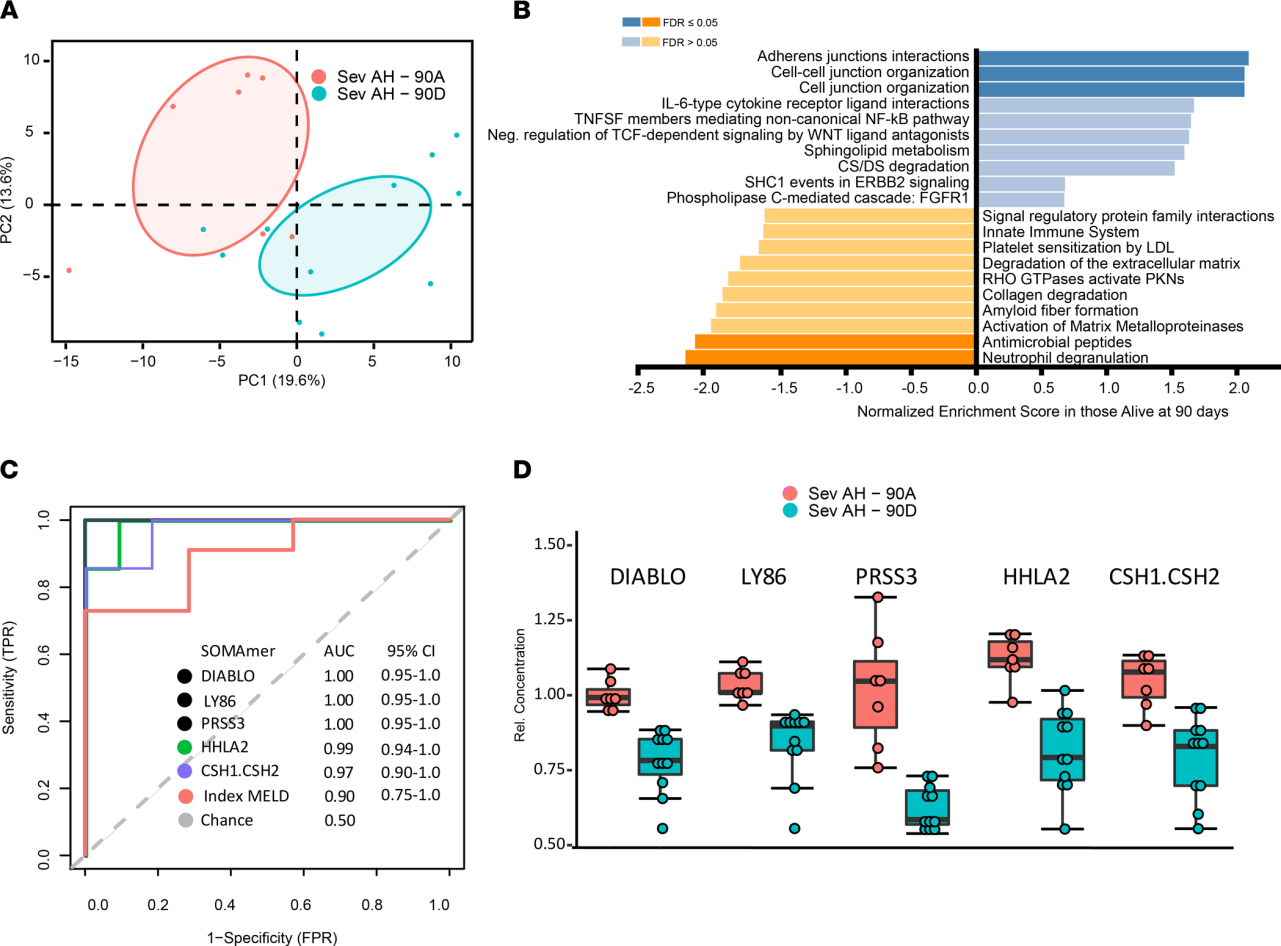
Analysis of proteins associated with 90-day transplant-free survival in severe AH. (**A**) PCA and (**B**) gene set enrichment analysis of patients with severe AH alive or dead or transplanted at 90 days. (**C**) Receiver operating characteristic curves of top 10 discriminating proteins as well as MELD. TPR, true positive rate; FPR, false positive rate. (**D**) Top 5 proteins distinguishing death at 90 days. Data are shown as mean ± SEM.

The proteomic signature of transplant-free survival versus the proteomic signature of occurrence of transplant or death 90 days after a diagnosis of severe AH were different (Figure 4A), and gene set enrichment analysis (11) highlighted gene sets associated with antimicrobial responses (Figure 4B), such as neutrophil degranulation and antimicrobial peptides. To identify potential biomarkers of 90-day transplant-free survival with severe AH, we applied receiver operating characteristic analysis to clinical and proteomic data. In line with previous data, the index MELD score was excellent at predicting 90-day transplant-free survival (AUC 0.90, Figure 4C). We identified multiple proteins that exceeded MELD’s prediction of 90-day mortality, the top 5 of which included DIABLO, a regulator of apoptosis; LY86 (also known as MD-1), a lymphocyte antigen; and PRSS3, an isoform of trypsinogen (Figure 4, C and D).

## Discussion

ALD, and in particular AH, has long been appreciated as a disease state characterized by pronounced disturbances in hepatic and systemic physiology, which is clearly illustrated in multidimensional data, such as those obtained through RNA-Seq (12) and metabolomics (13). Our current study extends these observations to the circulating proteome, where we find marked alterations in multiple circulating proteins associated with ALD and in particular severe AH.

Our data identify hundreds of proteins associated with ALD severity and outcome, which may serve as useful biomarkers or be investigated as potential therapeutic targets. A particularly notable finding is the association of GDF15 with ALD, which emerged as one of the strongest upregulated proteins in severe AH and correlated well with disease stage. GDF15, a distant member of the TGF-β superfamily, has emerged as a strong biomarker in multiple other pathological or physiological states, such as mitochondrial disease (14), liver fibrosis (15), myocardial infarction (16), aging (17), and nonalcoholic steatohepatitis (NASH) in a recent SomaScan study (7), but to our knowledge has not yet been linked clinically to AH.

GDF15 is a potent hepatokine with pleiotropic tissue and systemic effects that has been demonstrated to be secreted in response to activation of the integrated stress response, a pathway recently shown to be activated in patients with AH and that may underlie the elevation of GDF15 in our cohort (18). Interestingly, in patients with cancer, cachexia and weight loss — which are also common clinical features of patients with severe AH — are associated with GDF15 (19), and in mouse models, anti-GDF15 antibodies reverse cancer cachexia (20). This suggests a possible role for GDF15 in certain clinical features of AH.

We also identified THBS2 as among the strongest predictors of ALD stage, and THBS2 was recently reported as a biomarker of NASH as well as advanced fibrosis (21). This suggests it may have a broad role in liver disease, reflecting shared pathology underlying both NASH and ALD, and adds to the growing evidence of substantial overlap between nonalcoholic fatty liver disease and ALD pathophysiology. Further investigation into the role of THBS2 in liver disease is therefore warranted.

The fact that nearly half of the proteins we measured were altered in severe AH has implications for the ability to interpret the clinical significance of a single cytokine or protein in ALD. Proteomic dysregulation is so pervasive in severe AH that there is a reasonable chance that any given protein will have significant differences in patients with severe AH, which should proportionally increase the burden of proof that a particular protein is relevant to the pathophysiology of AH. That being said, we anticipate this proteomic survey of ALD will find utility in the identification of potential biomarkers for both disease progression and potential novel therapeutic targets.

Although severe AH is often treated as a distinct clinical entity among patients with heavy alcohol use (22), our proteomic data suggest it is a severe manifestation of an underlying pathophysiological process present to various degrees in all stages of heavy alcohol use and is detectable in patients with heavy drinking without clinically apparent liver disease and normal liver test results. We suggest it is more likely that the distinct aspects of severe AH presentations relate to manifestations of hepatic decompensation (e.g., jaundice, ascites) in the face of severe hepatic injury rather than reflecting a distinct pathophysiological process. If true, the proteomic signature of AH might find utility as a marker for prognostication in patients who do not yet have severe liver disease or in testing medical therapies at the earliest stage of ALD, rather than only in patients with severe AH and already high mortality. A notable caveat is that although we can identify the proteomic signature of AH in patients with AUD and normal liver test results, only a fraction of these patients will develop overt liver disease and AH. We are unable to identify the presence of subclinical liver disease, such as mild fibrosis or steatohepatitis, in our AUD group. Our proteomic signature is therefore unable to identify those at risk for the development of progression to overt liver disease and AH, which is likely mediated by mostly undefined biological factors. Future prospective studies identifying proteomic signatures in heavy drinkers that predict progression to overt disease would be particularly informative.

Our study suffers from a number of other limitations. First is its descriptive nature, which does not allow causal inferences to be made. For example, we cannot infer from these data whether an elevation in a particular protein represents a compensatory homeostatic mechanism, has a causal role in progression of the disease, or is elevated simply due to tissue destruction and release. Second, our relatively small sample size and limited number of outcomes in our severe AH cohort necessitate a validation study to discriminate proteins robustly associated with 90-day outcomes. Third, the SomaScan is by its nature biased, measuring only a specific subset of circulating proteins, and our analysis is therefore limited to this relatively small subset of proteins. Finally, individual proteins of interest identified in the SomaScan platform should be validated by a secondary assay if possible (such as ELISA) because ELISA remains the gold standard for protein concentration measurements and allows comparison to prior literature, where the bulk of protein measurements are made via ELISA.

Despite these limitations, our work demonstrates proteomic dysregulation as a cardinal feature of AH, identifies multiple potential new biomarkers and potential new therapeutic targets for AH as well as an early proteomic signature for AH, and, we anticipate, will serve as a useful resource for further prognostic and therapeutic studies aimed at improving treatment of AH.

## Methods

### Human patients.

Patients were recruited between 2019 and 2020 from both the outpatient gastrointestinal clinic and the inpatient services at the Massachusetts General Hospital. Prespecified inclusion criteria were a) a diagnosis of AUD made as part of established clinical care by an internal medicine physician with subspecialty training in addictions medicine for all patients except healthy controls and b) the ability to provide informed consent. The AUD diagnosis was based on guidelines presented in the *Diagnostic and Statistical Manual of Mental Disorders*, fifth edition (DSM-5). Prespecified exclusion criteria were a) patients with evidence of other forms of liver disease based on laboratory values or chart review by a hepatologist, b) those being cared for in the intensive care unit, and c) those with a history of liver transplantation. Once a potential patient was identified, the inclusion and exclusion criteria were reviewed independently by 2 hepatologists not involved in the care of the patient. Basic clinical (e.g., laboratory values) and demographic data (e.g., age, sex) were obtained at the time of enrollment by review of the medical chart by 4 independent reviewers. Additional clinical outcomes such as death or liver transplant were obtained retrospectively and reviewed by 2 hepatologists not involved in the care of the patient.

Patients were categorized into clinical groups based on the following criteria: a) controls: healthy controls without a diagnosis of AUD based on criteria established in the DSM-5; b) AUD: patients with a diagnosis of AUD as specified above but with normal liver test results and no evidence of overt liver disease based on available imaging or physical exam conducted by a hepatologist; c) AH: patients with a diagnosis of AUD and hepatitis based on elevation of serum aminotransferases, but without evidence of cirrhosis based on available laboratory values, imaging, physical exam, or biopsy, where the severity of AH was categorized based on MELD as mild (MELD less than 10), moderate (MELD 11–20), or severe (MELD greater than 20). Patients with a diagnosis of severe AH conformed to the diagnostic definition of probable alcoholic hepatitis as defined by the NIH National Institute on Alcohol Abuse and Alcoholism (1); d) alcohol-associated cirrhosis: patients with an established history of cirrhosis based on available imaging or biopsy as determined by 2 hepatologists not involved in the care of the patients. Initial analyses were performed in a discovery cohort of 94 patients to generate candidate biomarkers and models. An independent cohort of 14 patients containing healthy controls and patients with severe AH was used for validation.

### Proteomics profiling.

Venous blood samples were drawn at enrollment, immediately after which serum was obtained and stored at –80°C. The median time from hospital presentation to sample collection was 4 days. Proteomics analysis was subsequently performed using the aptamer-based, proteomic SomaScan platform as previously described (23). Anomalous values defined as a *z* score greater than 4 were replaced with the mean value of that group. A MELD of 7 was assumed for graphical representation of control patients (e.g., Figure 2A), who did not have available laboratory values to calculate MELD.

### ELISA protein measurement.

Select measurements generated from the SomaScan analyses were validated via ELISA in both the initial cohort of healthy controls and severe AH and in an independent validation cohort. ELISAs from R&D Systems, Bio-Techne (DGD150 for GDF15, DST200 for IL1RL1, DFLRG0 for FSTL3, and DTSP20 for THBS2), were performed per the manufacturer’s instructions.

### Statistics.

Data were compared by 1-way ANOVA, 1- or 2-tailed Student’s *t* test, or Mann-Whitney *U* test as described. Gene set enrichment analysis was performed using WebGestalt (11). All figures and analyses were generated using R version 4.1.0 unless otherwise stated. The box plots depict the minimum and maximum values (whiskers), the upper and lower quartiles, and the median. The length of the box represents the interquartile range. *P* < 0.05 was considered statistically significant.

### Study approval.

Informed consent in writing was obtained from each patient, and the study protocol conformed to the ethical guidelines of the 1975 Declaration of Helsinki, as reflected by approval by the Massachusetts General Hospital institutional review committee.

## Author contributions

RPG, EAS, and JL designed and supervised the study. AGLV acquired data. RPG drafted the manuscript and performed initial data analysis, which was critically revised by RPG, EAS, and JL. All authors had access to all study data, take responsibility for the accuracy of the analysis, and approve the manuscript.

## Supplementary Material

Supplemental data

Supplemental table 1

## Figures and Tables

**Table 1:**
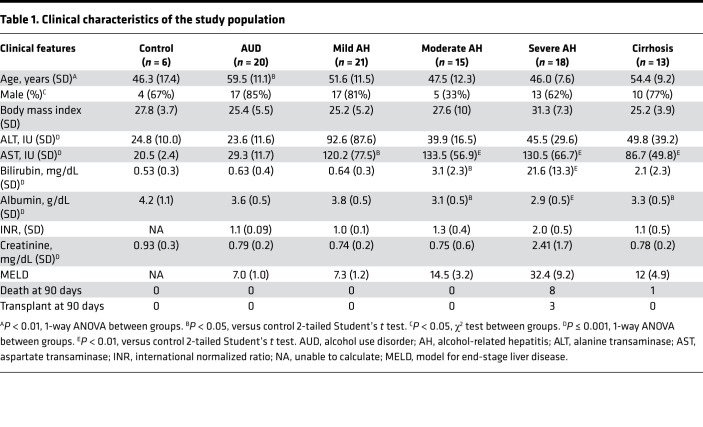
Clinical characteristics of the study population

**Table 2:**
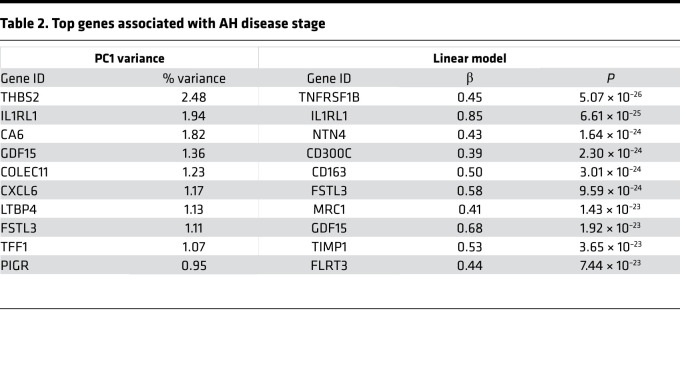
Top genes associated with AH disease stage

## Version 1 07/22/2022

Electronic publication
