# Supplementary material for: The circulating proteomic signature of alcohol-associated liver disease
Source: JCI Insight. 2022 Jul 22;7(14):e159775. doi: 10.1172/jci.insight.159775 (PMC9431701; doi:10.1172/jci.insight.159775)
Supplement: Supplemental data [file jciinsight-7-159775-s092.pdf]

Supplementary Figure 1

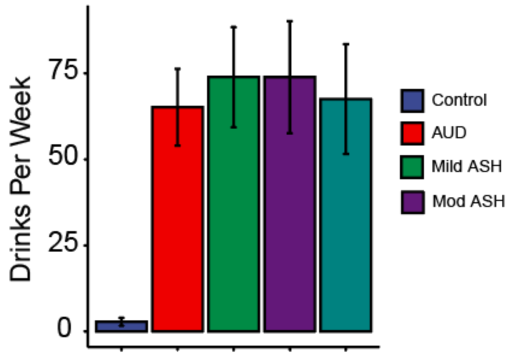

Supplementary Figure 1: Self reported standard drink consumption per week by diagnostic group. Data are mean +/- standard error of the mean.

Supplementary Figure 2

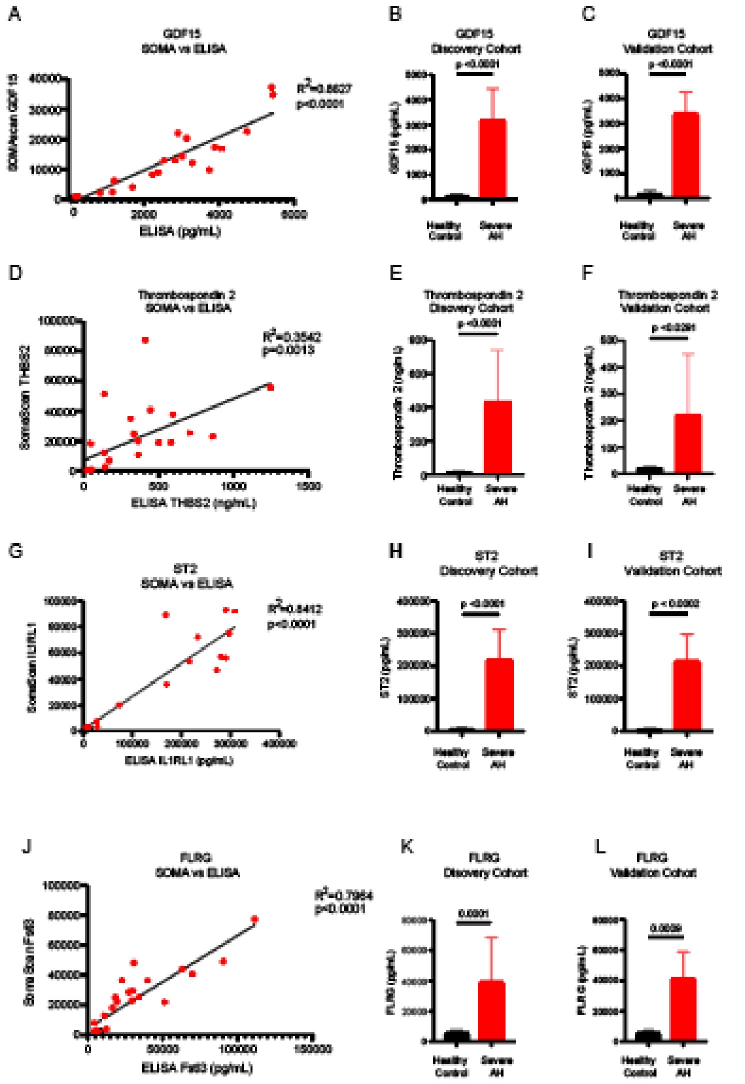

Supplementary Figure 2: Validation of top SOMAmer's associated with ALD. Correlation between SOMAscan and ELISA, and discovery and validation cohort ELISAs for GDF15 (**A-C**), Thrombospontin 2 (**D-F**), ST2 (**G-I**), FLRG (**J-L**). Data are mean +/- SEM.
